# Biopsychosocial Determinants of Cognitive Health in Later Life: The Harmonised Cognitive Assessment Protocol

**DOI:** 10.1093/geroni/igaa057.2203

**Published:** 2020-12-16

**Authors:** Dorina Cadar, Jessica Abell, Carol Brayne, G David Batty, David Llewellyn, Andrew Steptoe

**Affiliations:** 1 University College London, London, England, United Kingdom; 2 Cambridge Institute of Public Health, University of Cambridge, Cambridge, England, United Kingdom; 3 University of Exeter Medical School, Exeter, England, United Kingdom

## Abstract

Despite strong evidence for a socioeconomic gradient in many health outcomes, including cognition, substantial gaps remain in understanding these disparities. We investigated the biopsychosocial mechanisms underlying the associations between socioeconomic status (SES) and later-life cognitive health using the Harmonised Cognitive Assessment Protocol (HCAP), a sub-study of the English Longitudinal Study of Ageing (ELSA) which comprises of 1,273 ELSA participants aged 65+. A latent g factor was derived using 12 tests covering a broad range of cognitive domains (memory, language, executive function, and psychomotor speed). We estimated direct and indirect pathways between SES indicators, Apolipoprotein E, inflammatory markers, chronic conditions, and depression. We found that higher education was associated with better cognition, while wealth was not. Increased depressive symptoms were linked with lower cognition, while prior inflammation was indirectly associated with cognition via depressive symptoms and chronic conditions, supporting evidence for a psychosocial role in the context of a socioeconomic gradient.

